# The information challenge in public health crises: a study on the reliability and readability of information provided by large language model for thunderstorm asthma

**DOI:** 10.3389/fpubh.2026.1776697

**Published:** 2026-02-18

**Authors:** Zhenliang Zhu, Yanghui Feng, Feng Cao

**Affiliations:** 1 School of Nursing, Zhejiang Chinese Medical University, Hangzhou, China; 2 Department of Critical Care Medicine, Zhejiang Hospital, Hangzhou, China

**Keywords:** information response, large language model, readability, reliability, thunderstorm asthma

## Abstract

**Background:**

The advent of LLM (large language model) has seen extensive application in health information consultation, enabling interactive responses to complex queries; however, their reliability and readability warrant further investigation. This study aims to assess the reliability and readability of cross-disciplinary responses generated by artificial intelligence platforms regarding thunderstorm asthma, including ChatGPT-4, Deepseek-V3.2-V3.2, Perplexity Pro, and Microsoft Copilot.

**Methods:**

This study uses Google Trends to identify and filter topic-specific information on thunderstorm asthma. This study analyses cross-disciplinary responses generated by ChatGPT-4, Deepseek-V3.2, Perplexity Pro, and Microsoft Copilot in response to conversational inputs. The 29 selected responses exhibit varying levels of meteorological forecasting accuracy concerning thunderstorms, as well as prevalent themes related to asthma symptomatology and therapeutic interventions. The study employed reliability assessment tools, including the DISCERN instrument, the Ensuring Quality Information for Patients Scale (EQIP), the JAMA benchmarks, and the Global Quality Scoring (GQS), in conjunction with six authoritative readability metrics—namely, the Automated Readability Index (ARI), Coleman-Liau Grade Level (CL), Flesch–Kincaid Grade Level (FKGL), Flesch Reading Ease Score (FRES), Gunning Fog Index (GFI), and SMOG—to enable a comprehensive evaluation.

**Results:**

Research findings indicate statistically significant differences in the reliability of various artificial intelligence programmes when responding to complex interdisciplinary information queries. Microsoft Copilot demonstrates superior performance in terms of information reliability and structural quality, consistently achieving higher scores than ChatGPT-4-4o and Perplexity Pro, thereby providing more dependable information. However, all programme-generated informational responses were excessively complex for the general public, failing to meet sixth-grade reading comprehension standards, as the majority of outputs were written at a secondary education level or higher.

**Conclusion:**

This study reveals that while LLM demonstrate some reliability in handling complex health consultations, none meet the recommended readability benchmark for a sixth-grade reading level. Future efforts should focus on improving the reliability and readability of LLM generated health information to enhance comprehension amongst broader audiences.

## Background

Thunderstorm asthma represents a public health phenomenon triggered under specific meteorological conditions. Thunderstorms increase the concentration of airborne allergens in circulating air masses, leading to acute exacerbations or onset of asthma characterised by respiratory distress in susceptible populations. The majority of cases occur in the 30–50 age group, with onset predominantly during spring and early summer. Between 36 and 56% of affected individuals had no prior asthma diagnosis.

Since the initial documentation of thunderstorm asthma events in Birmingham, UK, in the early 1980s (1), globally, nearly 30 thunderstorm asthma events have been reported, resulting in more than 7,000 hospitalisations and at least 18 fatalities (2–6). The thunderstorm asthma event that occurred in Melbourne, Australia, in 2016 had significant and far-reaching impacts (5). The sudden surge in cases overwhelmed healthcare services, severely compromising the delivery of medical care. Hospital admissions increased tenfold, ultimately resulting in ten fatalities attributable to thunderstorm asthma. Shorter durations of continuous event sequences are associated with a higher likelihood of abrupt outbreaks. Comparable meteorological conditions have created favourable conditions for pollen rupture and particulate dispersion, which are clearly evident as primary triggers in thunderstorm asthma events associated with environmental changes.

In the context of global climate change, the environmental challenges we face are becoming increasingly severe. In 2022, a government assessment report confirmed that climate change has contributed to increased pollen concentrations, accelerated dispersal rates, and a rise in extreme weather events, posing a direct threat to respiratory health, including asthma incidence (7).

In the current global context marked by an uneven distribution of limited medical resources and compressed timeframes (8), the emergence of LLM has diversified the sources of health-related information (9, 10). Artificial intelligence conversational applications, such as ChatGPT-4, Deepseek-V3.2, Perplexity Pro, and Microsoft Copilot, are now widely deployed. The multiplicity of information acquisition channels constitutes a potential source of health information, making the analysis of their reliability and readability critically important. The reliability of information affects audience cognition and decision-making regarding health behaviours. Meanwhile, optimising the readability of information to align with sixth-grade reading comprehension levels enhances audience understanding (11, 12).

However, certain limitations remain in addressing the occurrence, early warning mechanisms, and effective prevention and control measures for thunderstorm asthma. As an emerging public health incident, the prevention, effective containment, and reduction of mortality risks amongst vulnerable populations within a limited timeframe are of significant importance. Previous studies have utilised tweets from social media platforms such as Twitter to develop early detection systems for acute health events, including thunderstorm asthma (13).

This study aims to conduct an in-depth analysis of the following two research questions: (1) Reliability Analysis of Information Provided by LLM on “Thunderstorm Asthma,” and (2) Does the readability of information provided by LLM on “Thunderstorm Asthma” meet the recommended standards for health education materials?

## Materials and methods

### Question source and processing

To achieve comprehensive retrieval coverage, this study determines the standardised medical term for “Thunderstorm Asthma” through the Medical Subject Headings (MeSH) database. A theme is a collection of terminology that shares the same conceptual framework across languages and may encompass the most relevant information (14). Ensure the comprehensiveness and accuracy of the research. The following MeSH entry terms have been identified: “Thunderstorm Asthma”, “Asthma Thunderstorm”, “Asthma”, “Tornadoes”, “Asthma storm”, “Thunderstorm and Health”, “Thunderstorm Warning”.

Google Trends serves as a dynamic indicator of search behaviour and public interest over time (15). In this study, relevant standardised terms were input into the Google Trends system to capture, to the greatest extent possible, the global search trends related to “Asthma Thunderstorm,” thereby enabling the identification of the most relevant search queries associated with the “Asthma Thunderstorm” topic. To conduct a five-year longitudinal analysis (2020–2025) of global search patterns and public attention (parameters: Worldwide; All categories; Popular searches; time frame: September 2020 to September 2025). To make up for the limitations of search engine data, respiratory critical care experts excluded duplicate and irrelevant information. After a series of screenings, 24 representative results related to “Thunderstorm Asthma” were finally determined to be used for the model performance benchmark information assessment of the LLM (see Table 1).

**Table 1:** Google trends data, 25 keywords related to thunderstorm asthma worldwide from 2020 to 2025.

Thunderstorm asthma: (2020/09–2025/09, worldwide)	
Top	Relevance
1. Melbourne thunderstorm	100
2. Asthma Melbourne	99
3. Melbourne thunderstorm asthma	99
4. Thunderstorm warning	74
5. Asthma thunderstorm warning	74
6. Victoria thunderstorm asthma	44
7. Thunderstorm asthma today	31
8. Thunderstorm asthma warning Victoria	31
9. What is “thunderstorm asthma”	23
10.what is “asthma”	23
11.what is “thunderstorm”	23
**12. Pollen (removed)**	**22**
13. Thunderstorm asthma Australia	17
14. Melbourne weather	15
15. Asthma symptoms	15
16. Thunderstorm asthma symptoms	14
17. Melbourne weather thunderstorm asthma	14
18. Thunderstorm asthma warning today	14
19. Thunderstorm asthma warning Melbourne	13
20. Thunderstorm asthma forecast	12
21. Pollen count	11
22. Melbourne pollen	9
23. Asthma storm	8
24. Epidemic thunderstorm asthma	7
25. Thunderstorm asthma attack	6

To improve the systematic assessment, the final search results are summarised. This study selected four mainstream LLM, including ChatGPT-4-4o, Deepseek-V3.2, Perplexity Pro, and Microsoft Copilot (version: 1.240.110.0), to conduct a benchmark performance analysis on the quality of information responses provided by LLM. Large language model have a certain degree of exclusivity. Their internal weight information, training language databases, etc. are not made public. It is impossible to determine whether the training data sets of LLM contain relevant public health information on “thunderstorm asthma.” To avoid data contamination that may result from the opacity of data structures and prevent any potential bias risks, all browser-related data are cleared before each prompt word is input to prevent artificial intelligence from relying on cached data or internal algorithms from the previous interaction.

#### Reliability evaluation

To ensure a thorough assessment of the benchmark performance advantages of LLM, this study, based on an optimised process, is oriented towards non-professional patients, providing benchmark information response support without requiring users to have professional levels. Four most prominent LLM were chosen to be analysed in relation to particular aspects of the quality of information, namely DISCERN (content integrity), EQIP (presentation clarity), Global Quality Score (narrative coherence), and JAMA benchmarks (source transparency) evaluate the reliability of the benchmark performance of responses based on LLM.

The DISCERN instrument, developed in 1999 through a collaboration between the University of Oxford and the National Health Service (NHS) in the United Kingdom, is designed to assess the quality of information on treatment options provided by health-related websites (16). The instrument consists of three sections and contains 16 items. Scoring is interpreted as follows: a total score of 63–75 indicates excellent quality, 51–62 good quality, 39–50 average quality, 27–38 poor quality, and 16–26 inferior quality.

The EQIP tool evaluates the quality of documents on health information websites by assessing content, data identification, and structural features to measure their reliability, usability, and effectiveness (17). The instrument consists of 20 items, each rated on a four-point scale (“Yes,” “Partially,” “No,” “Not Applicable”). The EQIP score is calculated as a percentage, and the overall mean EQIP score is categorised into predefined quality classifications.

The GQS provides a comprehensive assessment of the quality of health-related content. The GQS employs a five-point scale to evaluate online health information on quality, information flow, and usability: 1 = inferior quality; 2 = poor quality; 3 = average quality; 4 = good quality; and 5 = excellent quality.

JAMA serves as the core framework for evaluating the appropriateness of professional health information, using descriptive metrics to assess authorship, sourcing, referencing, timeliness, and disclosure of conflicts of interest (18). Each factor is scored on a scale from 0 to 1, yielding a total score ranging from 0 to 4.

All evaluations were conducted independently by authors FC and ZLZ following a standardised procedure. ICC analysis was used to assess the consistency amongst all raters, ensuring the reliability of the final scores between raters (19). In the event of a disagreement, the senior researcher YHF will assess the final score.

#### Readability evaluation

Readability is assessed using six widely recognised indices—ARI, CL, FKGL, FRES, GFI, and SMOG—through an online calculator.^1^ Based on considerations of health literacy and patient safety, and to facilitate patient comprehension, the American Medical Association (AMA) and the National Institutes of Health (NIH) recommend that healthcare materials be written at a sixth-grade reading level (20). A FRES score of ≥80.0 indicates easily understandable text, while scores below 6.0 on other readability metrics indicate high comprehensibility.

Automated Readability Index (ARI)18:


4.71(characterswords)+0.5(wordssentences)–21.43

Flesch Reading Ease Score (FRES)18:


206.835–1.015〈/span〉(wordssentences)–84.6(syllableswords)

Gunning Fog Index (GFI)19:


0.4[(wordssentences)+100(complexwordswords)]

Flesch–Kincaid Grade Level (FKGL)19:


0.39(wordssentences)+11.8(syllableswords)–15.59

Coleman-Liau Index (CL)20:


5.89(characterswords)–0.3(sentenceswords)–15.8

Simple Measure of Gobbledygook (SMOG)21:


14.430×polysyllables×30sentences+3.1291

### Statistical analysis

#### Reliability and readability analysis

This study employs a multi-dimensional assessment framework, including DISCERN, EQIP, JAMA, and GQS, to benchmark the performance of four LLM based response systems—ChatGPT-4-4o, Deepseek-V3.2, Perplexity Pro, and Microsoft Copilot (Version: 1.240.110.0). Given that the rating data did not conform to a normal distribution, the statistics were described by the median and interquartile range (IQR), and the non-parametric Kruskal-Wallis test was used to compare the statistical differences in reliability scores amongst different LLM.

In readability analysis and assessment, readability scores for each LLM information responses are calculated based on six readability metrics: Automated Readability Index (ARI), Coleman-Liau Index (CL), Flesch–Kincaid Grade Level (FKGL), Flesch Reading Ease (FRES), Gunning Fog Index (GFI), and Simple Measure of Gobbledygook (SMOG). Since the distribution of readability score data fails to meet the normality assumption required for parametric tests, the Kruskal–Wallis non-parametric test was also employed to examine statistically significant differences in readability scores across the four programmes.

### Comparison with 6th-grade benchmarks

The Wilcoxon signed-rank test was used to evaluate the information responses of LLM robots against the reading comprehension level of sixth graders. The readability scores generated by artificial intelligence were compared with the established reference values for sixth graders to analyse the statistical differences.

All tests were two sided statistical tests and the predetermined level of significance was *p* < 0.05. The data processing, statistical analysis, and visualisation were done using R (4.5.1).

## Results

### Reliability analysis

This study utilised the DISCERN, EQIP, JAMA, and GQS assessment tools to evaluate the reliability of LLM generated responses. The reliability index of each LLM programme is measured by descriptive statistics (median [Q1, Q3]) (see Table 2). The inter-rater intraclass correlation coefficient amongst all evaluators was 0.813 (0.767–0.864). In the model stability assessment, to ensure consistency in the generated informational responses and mitigate response randomness, all LLM bots were operated under deterministic parameters (temperature = 0.0) and executed three times independently. The results were entirely consistent, yielding a stability coefficient of 100%. The Kruskal-Wallis test results indicate statistically significant differences in DISCERN, EQIP, and JAMA scores across the different LLM systems. In contrast, no statistically significant difference was observed for GQS scores (*p* = 0.843).

**Table 2:** Reliability score of artificial intelligence programme information response.

Row type	DISCERN (Q1, Q3)	EQIP (Q1, Q3)	GQS (Q1, Q3)	JAMA (Q1, Q3)
ChatGPT-4_4o	40 (36.47)	65 (60, 65)	4 (3, 4)	1 (0, 2)
Microsoft Copilot	48.5 (46.5, 50)	70 (65, 71.25)	4 (3, 4)	2 (0, 2)
Perplexity_Pro	45 (42.75, 47)	62.5 (60, 65)	4 (3, 4)	1 (0, 2)
Deepseek-V3.2	47 (46, 50)	60 (60, 60)	4 (3, 4)	0 (0, 0)
P	<0.001	<0.001	0.843	<0.001
H	25.987	36.115	0.826	25.213

**Figure 1:**
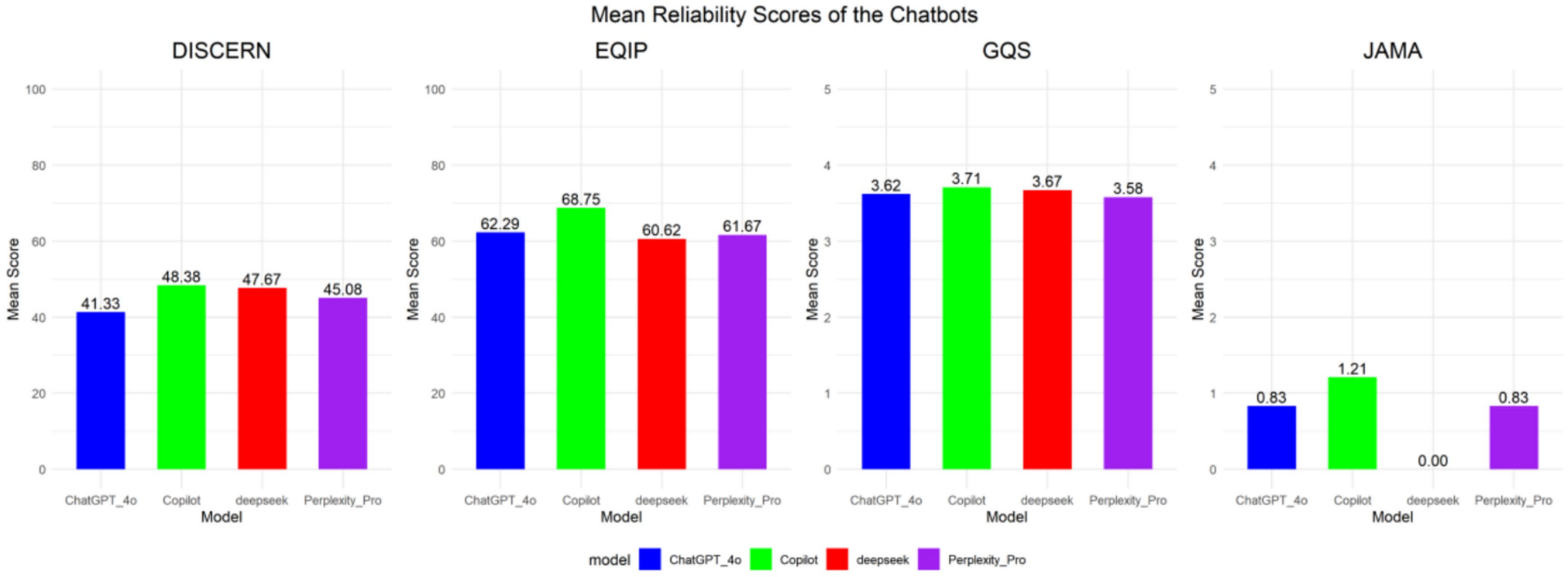
LLM programme response reliability ratings.

The DISCERN analysis reveals significant differences in the benchmark performance of information responses amongst various language models. The Perplexity Pro [45 (42.75, 47)] demonstrated a moderate level of performance, whereas ChatGPT-4-4o [40 (36, 47)] exhibited comparatively inferior information quality. This suggests significant variability in response reliability amongst LLM based conversational agents during information retrieval and analytical tasks. In contrast, the information responses generated by ChatGPT-4-4o exhibit a baseline performance of low quality, attributable to issues with source reliability, support capabilities, and decision-making processes. Although Microsoft Copilot [48.5 (46.5, 50)] and Deepseek-V3.2 [47 (46, 50)] received relatively higher scores and delivered moderately structured responses, their baseline performance is still rated as average overall (see Figure 1).

The EQIP results demonstrate that all evaluated LLM based agents received favourable quality assessments. Microsoft Copilot achieved the highest score [70 (65, 71.25)], while Deepseek-V3.2 received the lowest [60 (60, 60)]. All LLM based systems demonstrated satisfactory performance in terms of overall structure and clarity, with their comprehensive performance ratings evaluated as “good.” However, deficiencies were noted in the provision of detailed source information, textual readability, and balanced multi-perspective analysis (see Figure 1).

The GQS scoring results indicated no statistically significant differences. The performance of artificial intelligence in providing basic health information was moderately satisfactory, with an overall favourable impression, and the assessed LLM systems were rated as good (median = 4.0) (see Figure 1).

The JAMA rating scale revealed a statistically significant difference in outcomes, with Deepseek-V3.2 (median score: 0 [0, 0]) and Microsoft Copilot (median score: 2 [0, 2]) demonstrating distinct performance levels. The performance of LLM in adhering to evidence-based medicine standards reveals significant deficiencies in providing reliable author-related information and disclosures, particularly regarding authorship attribution and conflict-of-interest statements (see Figure 1).

To delineate the baseline performance disparities in information response capabilities amongst LLM, a Dunn’s test was employed for pairwise comparative analysis, thereby elucidating the inter-model performance variations (see Table 3).

**Table 3:** Test results of Dunn given in the form of reliability scores (*p*-values).

Comparison	DISCERN	EQIP	GQS	JAMA
ChatGPT-4_4o – Microsoft Copilot	0.000	0.000	1.000	0.219
ChatGPT-4_4o – Deepseek-V3.2	0.000	0.084	0.974	0.001
Microsoft Copilot – Deepseek-V3.2	0.522	0.000	0.949	0.000
ChatGPT-4_4o – Perplexity_Pro	0.201	0.547	1.000	1.000
Microsoft Copilot – Perplexity_Pro	0.004	0.000	1.000	0.274
Deepseek-V3.2 – Perplexity_Pro	0.023	0.228	1.000	0.002

On the DISCERN instrument for assessing information reliability and quality, ChatGPT-4-4o demonstrated significantly superior performance compared to Microsoft Copilot (*p* < 0.001) and Deepseek-V3.2 (*p* < 0.001). Meanwhile, Perplexity Pro demonstrated significantly superior performance compared to Microsoft Copilot (*p* = 0.004) and Deepseek-V3.2 (*p* = 0.023). However, no statistically significant differences were observed between ChatGPT-4-4o and Perplexity Pro (*p* = 0.201), nor between Microsoft Copilot and Deepseek-V3.2 (*p* = 0.522).

On the Evaluation of the Quality of Information Provision (EQIP) scale, the differences between the vast majority of model pairs did not reach statistical significance (*p* > 0.05).

In the evaluation using the Global Quality Score (GQS) scale, pairwise comparisons amongst all models did not reveal statistically significant differences (all *p*-values >0.999), indicating that the outputs of all models were perceived to be of comparable quality in terms of overall perceptual assessment.

In assessments conducted using the JAMA scale to evaluate information transparency, the performance of Deepseek-V3.2 was significantly inferior to all other models. By contrast, no statistically significant differences were observed in pairwise comparisons amongst ChatGPT-4-4o, Microsoft Copilot, and Perplexity Pro (all *p* > 0.05).

This study reveals that model performance is critically dependent on the selected evaluation framework. It further confirms the pervasive challenge faced by current LLM based systems in simultaneously ensuring the quality of structured information provision and adhering to conventional medical information transparency standards.

### Readability analysis

This study employs six widely recognised readability assessment tools: the ARI, FRES, Gunning-Fog, FKGL, Coleman-Liau, and SMOG indices. The median readability scores, presented as median (Q1, Q3) (see Table 4).

**Table 4:** Readability score of artificial intelligence programme response.

Programme (Q1, Q3)	ARIa	CL	FKGL	FRES	GFI	SMOG
6th grade level	6	6	6	80–90	6	6
ChatGPT-4-4o	12.78 (11.35, 15.62)	13.85 (12.45, 15.29)	10.37 (9.08, 12.97)	40.50 (36.50, 46.75)	11.65 (10.70, 12.70)	9.89 (8.16, 11.40)
Microsoft Copilot	12.45 (11.32, 13.30)	14.17 (12.88, 14.85)	10.15 (9.17, 10.76)	44.50 (39.00, 51.50)	11.20 (10.28, 12.30)	9.23 (8.72, 9.85)
Perplexity Pro	17.75 (15.19, 18.93)	16.48 (15.49, 17.26)	15.13 (12.72, 16.03)	27.00 (24.00, 36.25)	13.65 (12.18, 14.50)	12.57 (11.26, 13.45)
Deepseek-V3.2	13.52 (12.82, 15.41)	14.03 (13.16, 14.86)	11.40 (10.37, 12.05)	43.00 (36.75, 45.25)	11.90 (11.28, 12.73)	10.01 (9.49, 11.03)

Data are presented as median (interquartile range, IQR). The recommended scores for the 6th-grade reading level are provided as a reference. ARI, Automated Readability Index; CL, Coleman-Liau Index; FKGL, Flesch–Kincaid Grade Level; FRES, Flesch Reading Ease Score; GFI, Gunning Fog Index; SMOG, Simple Measure of Gobbledygook.

**Figure 2:**
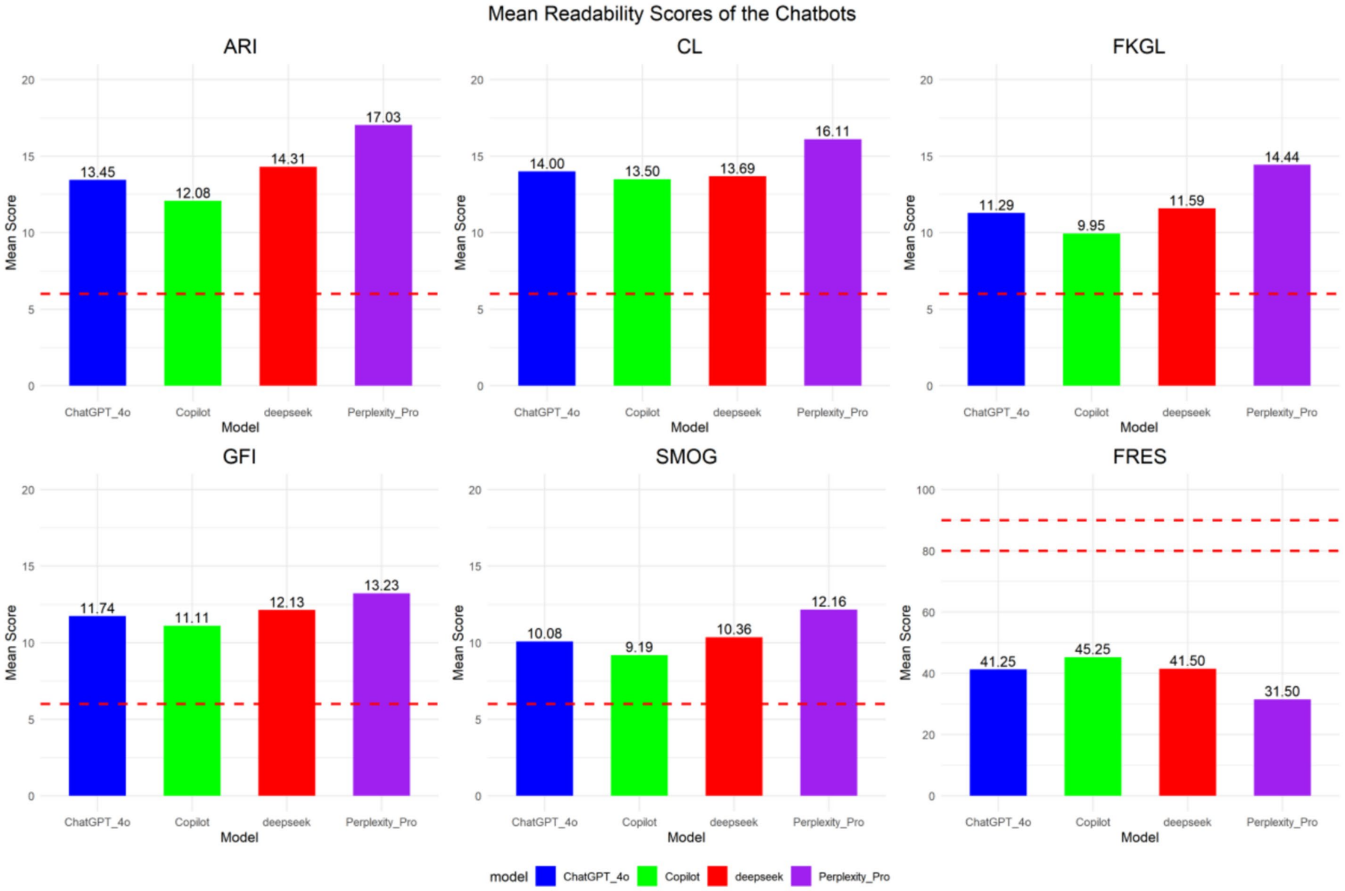
Mean readability scores of the LLM programme responds indexes. Red line indicates the 6th-grade level which is the highest recommended reading level for patient education materials. ARI, Automated Readability Index; GFI, Gunning Fog Index; FKGL, Flesch–Kincaid Grade Level; CL, Coleman-Liau Index; SMOG, Simple Measure of Gobbledygook; FRES, Flesch Reading Ease Score.

The results indicate that all analysed metrics for evaluating the readability of responses generated by major LLM —namely ARI, GFI, FKGL, CL, and SMOG—significantly surpassed the recommended benchmark for sixth-grade reading comprehension. The FRES score falls significantly below the benchmark range of 80–90 corresponding to the 6th-grade reading level. Even the top-performing Microsoft Copilot achieves a median score of merely 45.25, remaining within the difficult readability range. The results from ARI, GFI, FKGL, CL, and SMOG assessments collectively indicate that the text’s reading difficulty aligns with that of upper middle-grade levels. The standard information responses of the LLM robot evaluated in this study exhibit substantial readability challenges, with a comprehension level exceeding that of the general public (sixth-grade equivalent), thereby imposing certain limitations on vulnerable populations with low digital health literacy (see Figure 2).

## Discussion

With the advancement of artificial intelligence technologies, these systems have garnered increasing attention and adoption. Some users are now employing LLM as substitutes for traditional search engines to seek health-related advice or support decision-making in the healthcare domain. It is noteworthy that, compared to other types of informational responses, the reliability of medical and health-related information is of paramount importance, as erroneous content may lead to adverse health outcomes (21, 22). The findings of this study indicate that the information provided in the responses lacks critical details on certain key topics, exhibits moderate reliability, poor readability, and inconsistent quality. The quality of responses provided by LLM should be continually evaluated, where quality is defined as “the totality of characteristics of an entity that bear on its ability to meet stated and implied requirements” (23, 24). Findings from this study and other research indicate that the provided informational responses lack critical content in key subject areas and exhibit inconsistent performance across reliability and readability evaluation metrics.

### Reliability of LLM generated health information

This study employs the DISCERN instrument, EQIP, GQS, and JAMA benchmarks to evaluate the reliability and quality of health information responses regarding “thunderstorm asthma” generated by LLM. By leveraging the respective strengths and relevance of these four distinct assessment tools, a comprehensive, multi-faceted evaluation of response reliability and information quality is conducted (25). DISCERN and EQIP are widely used to assess the reliability and quality of written health materials. The former emphasises explicitly optimal evaluation criteria for studies on treatment efficacy, focusing on health information relevant to treatment decisions—including therapeutic benefits, risks, and quality of life outcomes (26). The latter focuses on the characteristics of information content in clinical application contexts. The GQS evaluation tool provides a subjective assessment of overall quality based on users’ individual needs and perceptions, as well as the universality of the information. JAMA evaluates four essential characteristics based on the standards for internet healthcare information published in the Journal of the American Medical Association, including authorship and attribution (e.g., author names and affiliations, cited references, copyright information), currency (e.g., date of posting, date of update), and disclosure (e.g., conflicts of interest, sponsorship, advertising, and commercial funding) (27).

The evaluation conducted through DISCERN, EQIP, GQS, and JAMA benchmark analyses indicates that Microsoft Copilot exhibits superior performance in information response compared to other LLM. The transparency and precision of the information sources utilised by Microsoft Copilot contribute significantly to its favourable reliability assessment. Meanwhile, ChatGPT-4’s reliability is compromised by the lack of source attribution and insufficient disclosure of information origins, whereas Deepseek-V3.2 demonstrates significantly lower scores on reliability and source transparency metrics—particularly under the DISCERN and JAMA evaluation frameworks—compared to other LLM systems. When seeking health information through LLM, internet users should prioritise the reliability of response sources, as the scientific rigour and evidence-based support of these sources have become increasingly critical indicators for assessing the credibility of health-related information, Incomplete dissemination of information and the provision of unreliable content may lead to harmful consequences, such as delays in seeking medical assistance or the adoption of erroneous preventive measures during similar public health emergencies. The transparency of information sources determines perceived reliability in health communication, whereas the dissemination of incomplete or misleading information undermines this credibility (19). The use of social media as a source of health information carries the potential risk of disseminating incomplete or misleading content (28).

### Readability of LLM generated health information

This study employs the ARI, CL, FKGL, FRES, GFI, and SMOG indices to evaluate the complexity of health information responses concerning “Thunderstorm Asthma” generated by LLM, thereby assessing the readability of these informational replies. Relevant research indicates that educational disparities are more likely to result in internet resources serving as a primary contributor to non-adherence to medical advice. Vulnerable populations, including old adults individuals and those with lower educational attainment, frequently encounter online health information that is highly readable yet unreliable. The development of health education materials tailored to the public’s reading level ensures that internet users can fully comprehend preventive measures for thunderstorm asthma and the safety of pharmacological treatments, thereby facilitating informed decision-making. High readability and reliable information responses are essential to prevent the dissemination of misinformation and enable the public to make informed preventive decisions regarding public health safety issues, such as thunderstorm asthma. Although studies indicate that both SMOG and GFI are more suitable as optimal indices for evaluating the readability of online health-related materials (29). However, for complex public health emergencies such as thunderstorm asthma, this study aims to assess the readability and complexity of LLM generated health information using multidimensional indices to yield comprehensive evaluation outcomes (30–32). The findings of this study indicate that the readability of all artificial intelligence programme-generated responses exceeds the recommended sixth-grade reading level, posing significant challenges in conveying health information to individuals with limited eHealth literacy. This study employs a multi-index evaluation framework to identify deficiencies in the content, structure, and other aspects of health information consultations provided through LLM question-answering systems. For instance, the Perplexity model’s responses regarding “thunderstorm weather forecasting” included statements such as “require carrying emergency medications” and descriptions about “symptoms and treatments of thunderstorm asthma”. The text includes the specialised terms “inhaled corticosteroid medications” and “airway spasm”; these ambiguous expressions and domain-specific terminology pose significant challenges for non-expert users in comprehension and information navigation. Science communication should employ accessible language that aligns with public needs, such as describing the mechanism as “alleviating airway hyperresponsiveness through inhaled aerosolised medications, exemplified by corticosteroids such as Budesonide,” to convey information clearly and concisely.

Currently, LLM can provide us with a vast amount of convenient information and have shown good performance in numerous studies. However, in complex interdisciplinary fields, especially in the health domain, the difficulty of responding to information may be too high for users with low e-health literacy, making it hard for them to understand and equally access key information. This is a problem that needs to be addressed at present. At the same time, when it comes to obtaining external information, if the retrieved information is from non-medical authoritative sources, the quality of such information cannot be guaranteed.

### Limitations

This study is subject to certain limitations. First, the exclusive reliance on Google Trends as a data source limits access to search query topics from alternative search engines, including regions affected by internet censorship or limited connectivity (33). Our global-scale analysis seeks to account for regional variations in thematic inquiries, linguistic diversity, and cultural differences, all of which may influence research outcomes. Currently, Google Trends remains the primary tool for analysing global trends in online health information searches. Furthermore, artificial intelligence-based LLM are constrained by limited sample sizes and are applicable only for interpreting probabilistic emergent events, such as thunderstorm asthma.

This study is based solely on a limited set of query data, which may not fully capture the broad spectrum of user needs concerning thunderstorm asthma health information. The scope of the training data, potential computational biases, and other inherent limitations may affect the accuracy, completeness, and clarity of the generated responses.

The readability and reliability metrics used in this study may not fully capture the nuanced distinctions inherent in LLM generated content. While evaluation results for various indices indirectly reflect information quality, they do not account for subjective factors, such as users’ contextual needs.

In the future, LLM systems will undergo continuous iteration and refinement. The evaluation of LLM generated responses in healthcare information must be regularly updated accordingly. Furthermore, it is essential to explore real-time user interaction data to accurately assess the reliability, readability, and practical utility of health information provided by LLM applications.

## Conclusion

This study highlights the importance of dynamic evaluation in the response process of LLM for healthcare information inquiries. Although platforms such as Perplexity and Microsoft Copilot demonstrate acceptable reliability, their information readability remains suboptimal. Going forward, the development of LLM applications must prioritise addressing diverse users’ reliability requirements and linguistic readability needs, which are critical for facilitating effective communication of healthcare information.

## Data Availability

The original contributions presented in the study are included in the article/supplementary material, further inquiries can be directed to the corresponding author.

## Footnotes

1
https://readabilityformulas.com
